# Addressing Once More the (Im)possibility of Color Reconstruction in Underwater Images

**DOI:** 10.3390/jimaging10100247

**Published:** 2024-10-08

**Authors:** Yuri Rzhanov, Kim Lowell

**Affiliations:** Center for Coastal and Ocean Mapping, University of New Hampshire, Durham, NH 03824, USA; klowell@ccom.unh.edu

**Keywords:** underwater imaging, color correction, computational color constancy, color reconstruction ambiguity

## Abstract

Color is an important cue in object recognition and classification problems. In underwater imagery, colors undergo strong distortion due to light propagation through an absorbing and scattering medium. Distortions depend on a number of complex phenomena, the most important being wavelength-dependent absorption and the sensitivity of sensors in trichromatic cameras. It has been shown previously that unique reconstruction in this case is not possible—at least for a simplified image formation model. In this paper, the authors use numerical simulations to demonstrate that this statement also holds for the underwater image formation model that is currently the most sophisticated.

## 1. Introduction

The reflectivity spectrum of any object is its richest, and thus most unique, visual property. The species classification of marine life can be achieved most reliably through the accurate measurement of their reflectivity spectra. Unfortunately, such measurements require two pieces of expensive equipment—specifically, an instrument for the measurement of the illumination spectrum and a hyperspectral camera. It is intuitively clear that a conventional trichromatic camera cannot convey spectral information comparable to that conveyable by a hyperspectral (or even multispectral) sensor [1,2]. Thus, for a long time, the goal of researchers that used color information for classification purposes was to achieve reproducibility of color by eliminating the influence of external illumination and the specific spectral characteristics of the camera used [3]. A vast amount of the literature is devoted to various techniques for the reconstruction of color that would be recorded under certain known illumination conditions and with a camera with known parameters. Among these techniques, the most popular are white balancing [4] and dark channel prior [5,6,7].

Underwater imagery poses additional difficulties in terms of color reconstruction due to wavelength-dependent absorption and scattering by the propagation medium, suspended particles, and dissolved organic matter. Nevertheless, a number of publications have proposed algorithms that are claimed to be able to correct underwater images “as if” they were taken in the air by compensating for absorption and scattering effects (for example, [8,9,10,11,12]).

In some papers, the authors were careful about their wording, mentioning something like “Recovering correct or at least realistic colors of underwater scenes…” [13]. Thus, they did not exclude the possibility of recovering “true” colors. Serious doubts about whether this is possible in principle have been expressed [14,15]. In these studies, numerical experiments employing a simple color formation model underwater showed that multiple objects with visually different colors when recorded in air appeared to be the very same color underwater, although all the relevant parameters (medium characteristics, camera parameters, etc.) were the same. Despite these statements, papers proposing the reconstruction of the “true” colors of underwater objects continue to appear in the literature.

Many papers where the authors propose underwater image enhancement (as opposed to reconstruction) have been published [16,17,18,19,20,21,22,23,24,25,26,27,28]. However, the value of the reported results remains questionable, as it is difficult to estimate how close the enhanced colors are to the “true” ones.

A comprehensive survey of proposed techniques for the reconstruction of “true” colors and color enhancement can be found in [25,29].

A more comprehensive study of color reconstruction ambiguity, however, has been published in [30]. All the steps of the simulations in that study were carefully verified, although the model was still simplified:

- Light scattering was ignored;
- Constant illumination was assumed for all wavelengths;
- The quantum efficiency curves of the sensor were approximated by means of Gaussians using the same parameters;
- The reflectance spectra were represented as three rectangles—each in one of the three main parts of the visible spectrum (red, green, and blue)—except for the maximum wavelength.

Note that the approximation of reflectance spectra with a piece-wise function as described and used in [31] and depicted in [30] confirmed the previously reported results.

Repeated demonstrations that the recovery of the “true” colors of objects captured in images obtained through water is not possible have not, of course, eliminated efforts to develop methods that achieve this. Understandably, research on recovering “true” color has evolved and advanced—most notably with the development of a more advanced “image formation model”. The purpose of this paper is to explore the ability of the “advanced” image formation model to recover “true” color even though previous methods have been shown to be inadequate.

## 2. Image Formation Model

Since the emergence of early underwater image formation models [32,33], a more elaborate image formation model has been proposed [34,35], currently allowing for the best (closest to in-air imagery) reconstruction of images taken underwater. The corresponding paper considers light propagation in a scattering medium, the inherent and apparent optical properties of water, etc. For detailed discussion and radiance transfer equations, the reader is referred to the original work [34]. It is sufficient to say that in the present paper, we adopt the proposed revised model and repeat our numerical experiments to determine whether model modifications affect color reconstruction ambiguity and in what manner.

Two simplifications employed in the previous paper [30] were also used in the simulations presented in the present paper:

1. Quantum efficiency (QE) curves were assumed to have a Gaussian shape;
2. The spectra of reflectors were represented by piece-wise functions, as explained in the articles cited above.

**Figure 1:**
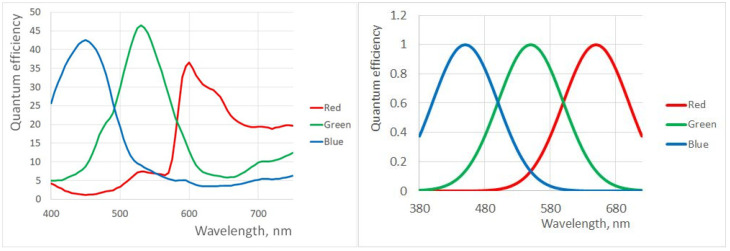
Quantum efficiency curves for GoPro cameras (data from open-access source, https://www.edge-ai-vision.com/wp-content/uploads/2021/01/Stern_GoPro_Embedded_Vision_Summit_Slides_Final.pdf, accessed on 3 October 2024) (**left**) and the (Gaussian) ones used in simulations (**right**). In both figures curves of different color represent QEs of three channels of a trichromatic camera.

The first assumption we consider to be viable, as many published QE curves indeed resemble Gaussians; compare the QE curves for GoPro cameras and those that were used in our simulations (Figure 1).

## 3. Results

The numerical experiments conducted are explained below.

Colors from the Macbeth chart were chosen as seeds, as they are de facto standard colors for colorimetric experiments. For each Macbeth color, the corresponding reflectance spectrum was found, with the conditions that illumination is constant for all wavelengths and absorption and scattering are absent (i.e., as if the image was taken in the air). Obviously, conceptually, there is a near-infinite number of reflectance spectra satisfying these conditions, and any particular solution can be found only through numerical optimization. We describe the adopted optimization technique here, as the same method was used throughout all the experiments described below. The software for numerical modeling was written in C++ by the authors.

Standard methods like conjugate gradients, Levenberg–Marquardt, CMA-ES [36], etc., are not suitable, as they involve finding the global (or local) minimum of the objective function, which is defined as the Euclidean distance between the target RGB value and the current one. In our optimization, we search for any solution that is sufficiently close to the target (i.e., the selected Macbeth color). Sufficiency in this context is understood as an inability to distinguish two colors recorded by modern digital sensors. The “closeness” of colors could be interpreted differently; in fact, if an RGB triplet examined and the target one lie in the same cube with all sides equal to 1, the color recorded by an 8-bit conventional camera will be the same as the target one. We, however, have set the threshold of “closeness” to $10^{-4}$.

In all simulations, the distance between the colored target and the camera was 3 m, and the depth at which measurements were taken was 1 m.

In the first step in each simulation, some color from the Macbeth chart is chosen (a seed color), and the resulting reflectance spectrum associated with this color in the air (with no absorption or scattering) is found using a random search, as follows.

Real spectral signatures have complex shapes, and using such shapes in numerical experiments is not practical as a large number of parameters are needed to define these shapes. Moreover, obtaining appropriate combinations of such parameters requires excessive searching in a very high-dimension space. Thus, we restricted spectral signatures considered to the same piece-wise representation that was used in previous works [30,31]. We believe that such a restriction does not affect this paper’s main result.

The search space has nine dimensions. For each channel, there is a “start” lambda (wavelength) and a “stop” lambda (the channel reflectance is zero for all wavelengths except for the region between these two values). Also, each such region has a certain “strength” of reflectance—i.e., the level of contribution to the final color. N1 random points are chosen in the search space with the following conditions: wavelengths vary in the 400–700 nm range, and regions of non-zero reflectance do not overlap. The point with the smallest value of the objective function is selected as the “best point.” Subsequently, the search region is reduced in size by some factor (0.3 was used in these experiments) around the best point, and a new set of random points is generated. This is repeated N2 times or until an objective function in one of the points is found to be less than the threshold shown above. In the former case, the result is ignored, and the whole process repeated. In our experiments, N1 had a value of 1000, and N2 had a value of 10, as these performed well.

For the simplified model reported in [30], the search occasionally (in fewer than 5% of iterations) stopped at a local minimum sufficiently far from the target to enable it to be ignored. For the revised model, however, all searches finished at a distance from the target that was less than the predefined threshold.

When the reflectance spectrum for the seed color was found, the equations from [34] were used to obtain its underwater counterpart (that includes light absorption and scattering). Due to arbitrary illumination intensity and an arbitrary reflectance spectrum scale, the resulting RGB triplet may not be in an 8-bit cube. If it is not, then color normalization is performed such that the maximum channel value does not exceed 255 and the color is not “too dark” (i.e., its triplet is visually indistinguishable from (0,0,0), although it represents entirely different chroma). The solution, including the reflectance spectrum and the RGB triplet, is saved in a file.

As mentioned above and explained in detail in [30], the considered reflectance spectra are represented by three rectangles positioned in “strategically” important parts of the visible spectrum—red, green, and blue. Thus, each normalized spectrum is defined by 9 parameters: the ranges of wavelengths where reflectance is nonzero and the relative strengths of the reflectance.

Next, the nine-dimensional space of the spectral parameters is randomly searched to find sets that, being different from the seed color set, lead to the same (or sufficiently close, as mentioned above) color underwater found previously. Obviously, due to the randomness of this procedure, there is no guarantee that all such sets can be found. However, even the existence of just two such sets demonstrates that knowledge of all medium parameters, the distance to the imaged object, and its color captured underwater does not allow for the unique reconstruction of a “true” color.

**Figure 2:**
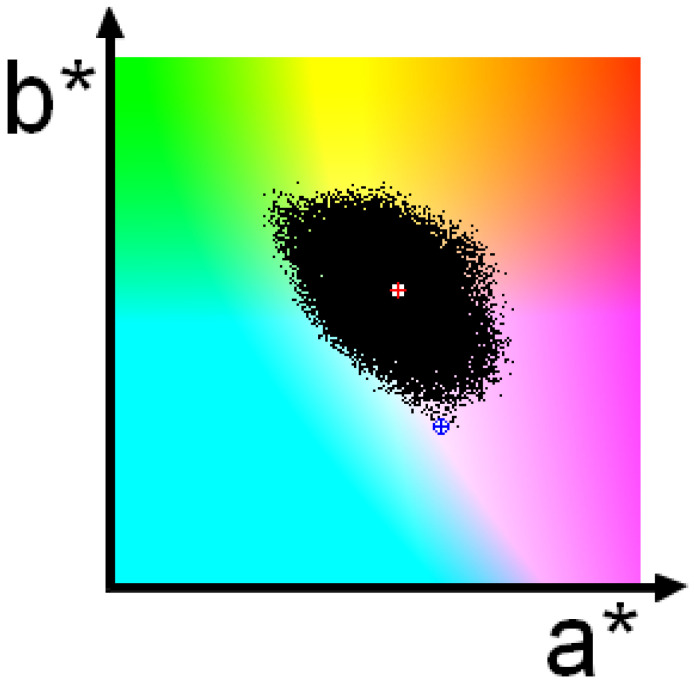
Each color “in air” that appears underwater as a selected “underwater” color is marked in this figure as a black dot in the (a*b*) subspace of the CIE L*a*b* color space. The red cross indicates the location of the “in air” seed color—the color arbitrarily selected from the Macbeth chart. The blue X in a blue circle represents the corresponding “underwater” color—the color that is recorded by a camera when it is pointed at the seed color.

Each set of parameters found using the procedure above was converted into a color in a CIE L*a*b* color space. The lightness component was set to 100 for convenience as it does not affect the chroma, and an L*a*b*-to-RGB conversion was performed. All the colors found were marked as black dots on a (a*b*)-colored subspace. Running the random search several thousand times, the area occupied by black dots reached near-stability. Figure 2 shows a (a*b*) subspace with such an area. A white circle with a red cross indicates the location of a seed color, and a white circle with a blue cross indicates the location of the corresponding underwater color.

**Figure 3:**
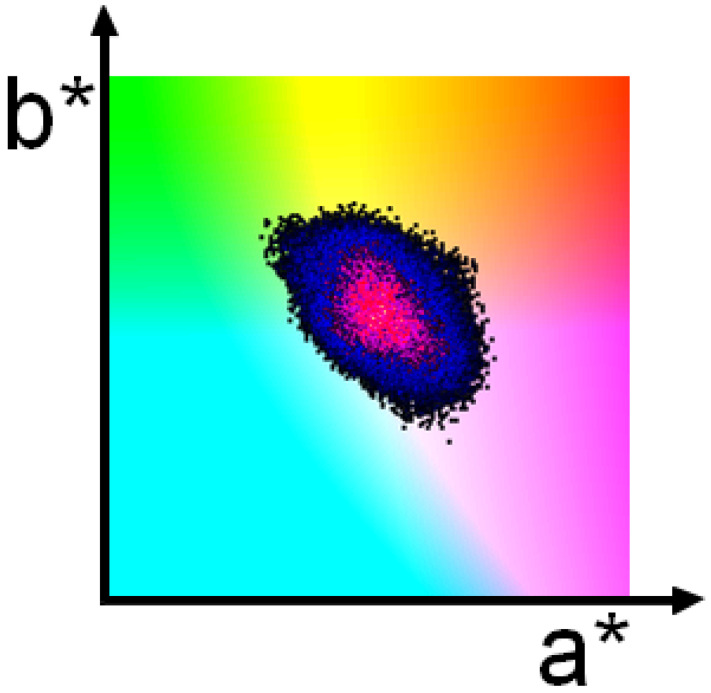
Orthogonal view of the frequencies of colors. The area with no found solutions (no black dots) is colored with (a*b*) subspace. In the area with found solutions, the black–blue–red–yellow colormap is used; black is used where the frequency of found colors is low, and yellow shows where the frequency is high.

**Figure 4:**
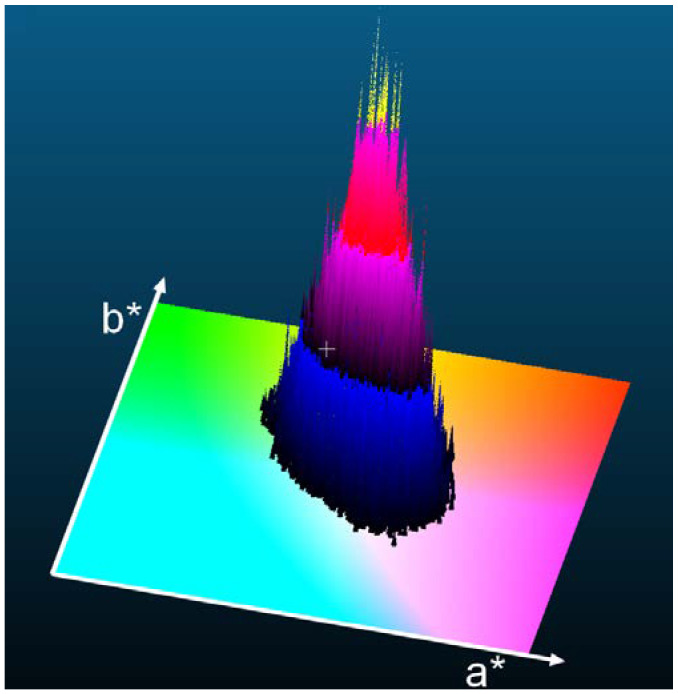
A 3D view of the frequencies of the colors found. The peak height has arbitrary scaling. The area with no found solutions (no black dots) is colored with (a*b*) subspace. In the area with found solutions, the black–blue–red–yellow colormap is used; black is used where the frequency of found colors is low, and yellow shows where the frequency is high.

Although the initial points in a nine-dimensional space were chosen uniformly within specified limits, the resulting solution is more likely to be found in certain areas than in others. The distribution is illustrated in Figure 3 and Figure 4. In Figure 4, the area without a found solution—i.e., the area with no black dots—is colored with (a*b*) subspace. In the area with found solutions, the black–blue–red–yellow colormap is used; black is used where the frequency of found colors is low, and yellow shows where the frequency is high. The (yellow) peaks in the frequency distribution are close to the seed color.

Thus, the previous results are re-confirmed: ambiguity remains even for the most accurate (to date) model for the formation of underwater images.

**Figure 5:**
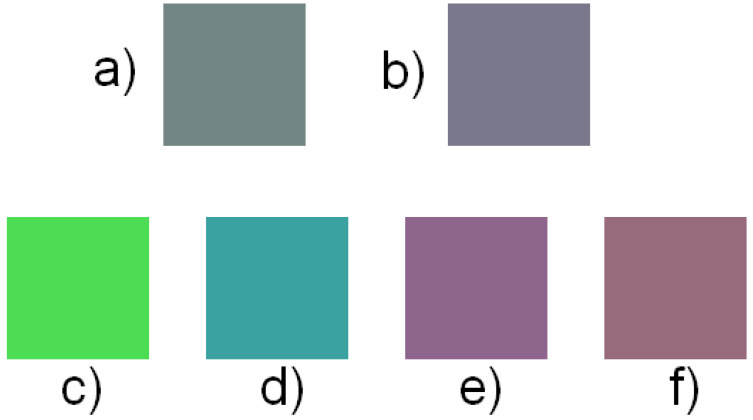
Recorded colors. (**a**) seed color, (**b**) same color underwater, and (**c**–**f**) colors recorded in air that also appear to be the color of (**b**) underwater.

To demonstrate the striking difference between in-air colors that appear as the same color underwater, sample solutions have been chosen from the opposite sides of the solution cloud shown in Figure 5.

**Figure 6:**
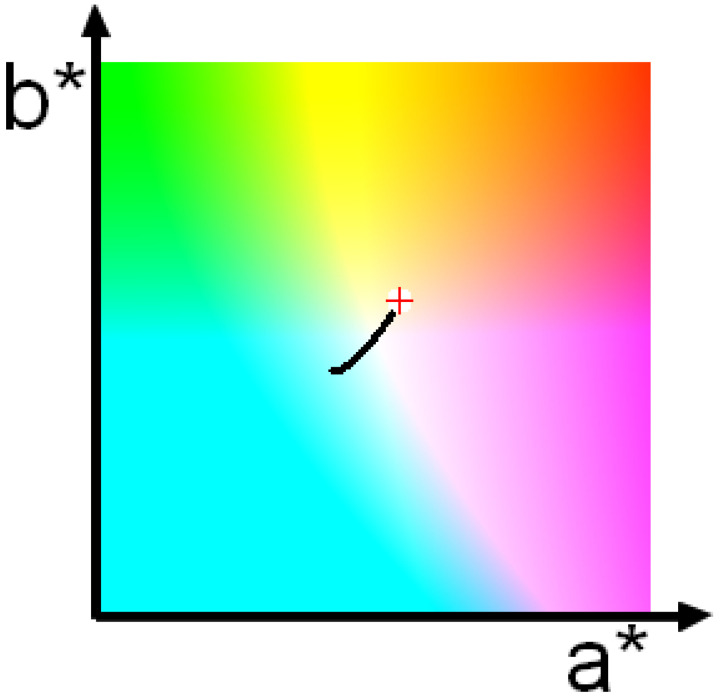
Change in recorded color due to change in range between the target and the camera. Red cross indicates the color recorded at zero range.

**Figure 7:**
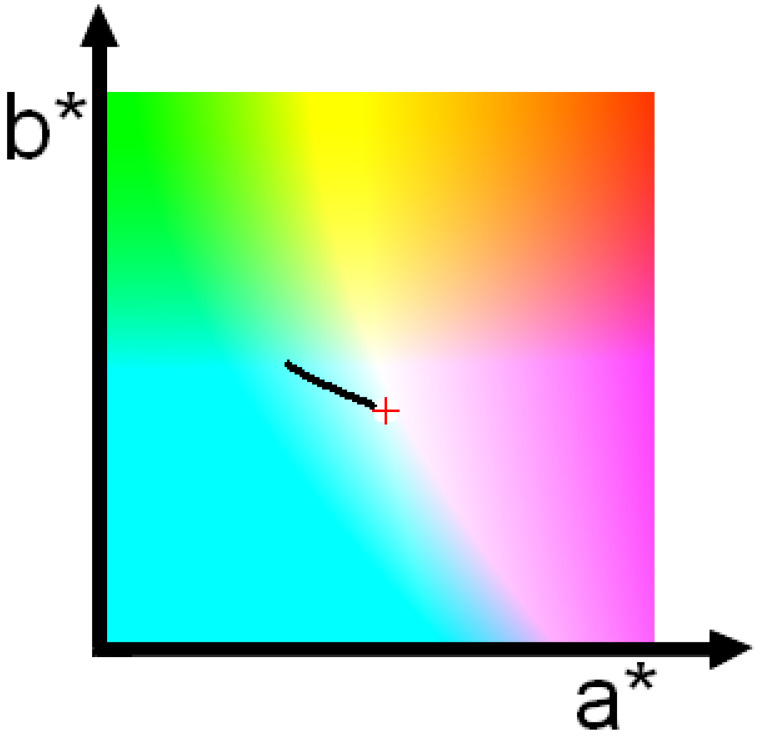
Another example of the color changing with the camera-target range. Red cross indicates the color recorded at zero range.

It was observed that the distance between the target and the camera strongly affects the recorded color. This is demonstrated in Figure 6 and Figure 7. The white circle with a red cross indicates the color taken at zero range, which is equivalent to a photo taken in the air for which absorption and scattering are negligible. When the range increases from 0 to 10 m, the color of the target changes, following the black curve in the left–down (Figure 6) of left–up (Figure 7) direction. In Figure 6, in the air, the target appears yellow, but with an increasing range, it becomes pink and then blue. In Figure 7, the target becomes more and more greenish. We would like to remind the reader that only changes in chroma are reflected; brightness is ignored.

## 4. Conclusions

The goal of the presented work was to examine if the recovery of “true” colors from underwater imagery acquired with a trichromatic camera and a more sophisticated, recently formulated image formation model is possible; the results indicate that it is not. Hence, any algorithms proposed to be able to correct colors in underwater scenes should be considered to be limited to the creation of “more realistic” colors or even the beautification of a scene. Moreover, the use of these colors for classification purposes may lead to serious mistakes, even if all the parameters of illumination, camera, and the propagation medium are known.

## Data Availability

The data and software can be provided by authors upon request.
